# Large hand mass due to poor primary care access in an internally displaced person in the DRC: a case report

**DOI:** 10.1093/omcr/omaf094

**Published:** 2025-07-14

**Authors:** Rory Marples, Bavon Baruti Murakirwa, Charles Luhombo Kataala, Phanny Kambere Simisi

**Affiliations:** Medecins Sans Frontieres, Goma, Democratic Republic of Congo; Medecins Sans Frontieres, Goma, Democratic Republic of Congo; Medecins Sans Frontieres, Goma, Democratic Republic of Congo; Virunga Hospital, Goma, Democratic Republic of Congo

**Keywords:** hand tumour, myxoma, surgery, general surgery, war and armed conflict, primary care

## Abstract

We report a woman in her twenties from a displaced persons camp in the Democratic Republic of Congo (DRC) who presented to our medical clinic with a large exophytic tumour from the palm of her right hand. Examination revealed a 100 x 80 x 50 mm tender, necrotic mass, with no associated lymphadenopathy or metastases. Excision and histopathological analysis confirmed the tumour as a myxoma. This case underscores the rarity of palmar myxomas and highlights the impact of armed conflict on healthcare access. Delayed diagnosis and treatment were attributed to disrupted local health infrastructure and poor access to primary care due to the ongoing conflict in the eastern DRC between the Congolese Army and the M23 paramilitary group. The case emphasises the critical role of primary healthcare in conflict zones for timely diagnosis and referral, which is needed to reduce morbidity from neglected surgical diseases.

## Case report

A woman in her twenties presented to a medical clinic in a displaced persons camp in the eastern DRC with a large tumour on the palm of her right hand. She reported that the mass had developed over the previous four months, and had grown to the point where it caused significant pain and affected the function of her hand. The tumour’s size and weight made it difficult for her to perform activities of daily living, severely impacting her quality of life. She had no significant medical history, allergies, or family history of cancer. The patient was right-hand dominant and had been forcibly displaced from her village by armed rebel groups about a year before presenting to the clinic.

**Figure 1:**
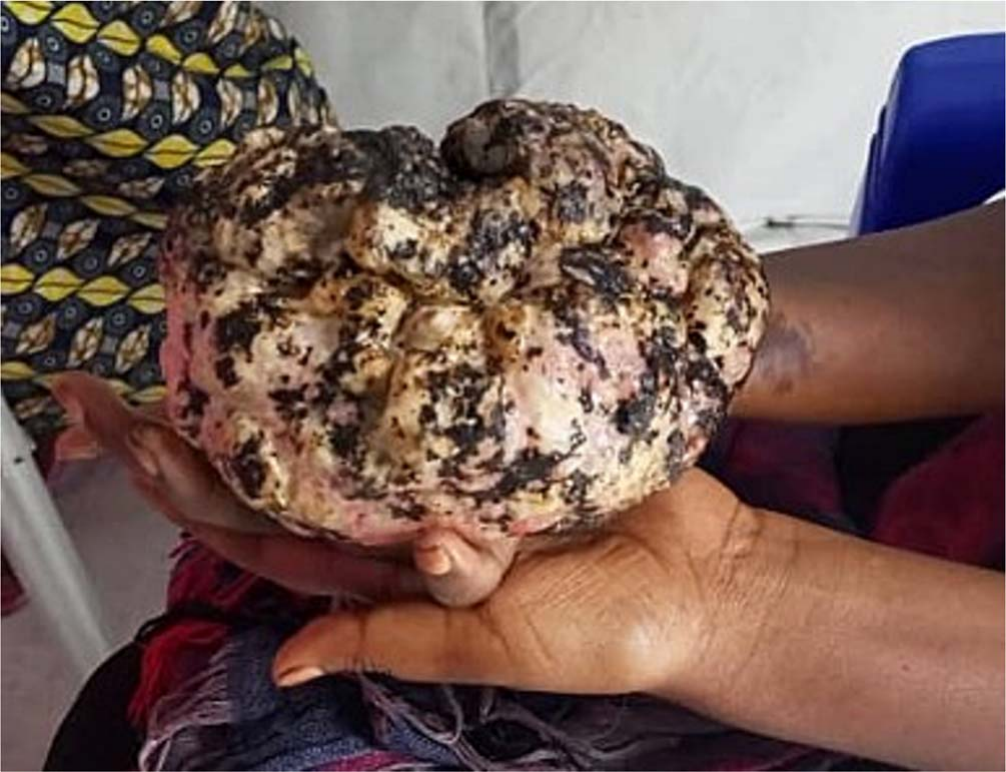
Clinical photograph of the exophytic tumour on the patient’s right palm, taken during the initial consultation, showing prominent ulceration and necrotic patches on its surface.

On physical examination, the patient had a large, tender, irregular, exophytic tumour on the palm of her right hand. The mass was ovoid in shape, measuring approximately 100 × 80 × 50 mm, and was tender to palpation around the base (Fig. 1). The tumour had displaced her fifth digit laterally, and she was unable to hold objects with her hand. Although grip and flexion strength could not be fully tested due to the size of the tumour, she was able to partially flex her fingers up to the limit of the mass. There was no sensory deficit to her hand or fingers. The tumour was malodorous and had necrotic patches on its surface, appearing heavy and requiring her to support it with her non-dominant hand or rest it on her knee. There were no signs of epitrochlear, axillary, or subclavian lymphadenopathy, and no tenderness was found on palpation of the axial skeleton, ruling out the possibility of distant metastases.

The patient was afebrile, and her vital signs were within normal limits. It appeared that she had not received prior treatment or been referred to a secondary care facility, although the presence of a crepe bandage around the tumour suggested that she had sought some form of first aid in the weeks prior to presentation. After initial evaluation, the patient was given paracetamol and ibuprofen for pain relief and was referred to a local hospital for further assessment and surgical evaluation. Due to resource limitations, no imaging studies were performed to assess deeper structures, vascular involvement, or potential metastatic spread, and she was admitted directly under the surgical team.

The following day, she underwent an en bloc tumour excision with primary closure of the wound, and the tumour was sent for histopathological examination. Intraoperatively, the lesion appeared to be attached to the connective tissue surrounding the tendons, though this was not definitively confirmed given the tumour’s size and extent, and there was no significant bleeding or damage to surrounding structures. The patient had a good recovery and remained in the hospital for rehabilitation, physiotherapy, and further pain management while awaiting her histopathology results.

Histological analysis of the excised tumour confirmed it was a myxoma. This was confirmed by the presence of tissue with benign ulcero-necrotic neoplasia, with fibroplastic, fusiform, non-mitotic starry cells within a myxoid framework. There was no indication of malignant transformation or tumour extension, and the margins of the excision were clear, confirming complete removal. At the time of writing, the patient was still receiving postoperative wound care and physiotherapy and remained an inpatient in the hospital.

## Discussion

Myxomas are benign tumours typically composed of stellate cells suspended in a myxoid stroma and are most commonly found in the heart [1]. However, extra-cardiac myxomas have been reported, and they are exceedingly rare in the hand [2]. Previous palmar myxomas have been reported as originating from the nerve sheath [3], intramuscularly [4] and from the juxta-articular region [5]. Despite efforts to completely excise these tumours, there remains a reported high rate of recurrence [2]. This is particularly important to note in low-resource and conflict settings due to the potential difficulties in arranging patient follow-up in the case of recurrence. Surgeons should be aware of this when planning operations for excisions of such lesion, for example considering long-term admission under histopathology results are obtained. Although its likely that the tumour originated from the palmar fascia, this was not confirmed intraoperatively or in the histopathology report, nor was there any mention of a juxta-articular origin. The exophytic nature of the tumour, with ulceration and necrosis on its surface, is unique in comparison to other reported cases.

This case is significant for two reasons. First, large exophytic hand tumours like this are rare, with few cases documented in the literature, and this appears to be one of the largest ever recorded. Hand tumours are often identified early, given the functional impairment they cause, especially in the dominant hand. In regions with well-functioning primary healthcare systems, patients can be quickly triaged and referred for specialized care, preventing such tumours from reaching a stage of considerable size. Unfortunately, the patient’s limited access to primary healthcare, due to the ongoing conflict, delayed the timely diagnosis and intervention. Clinicians working in these environments should be aware of more advanced presentations of disease and the need to treat them without availability of usual resources or investigations, such as blood tests, x-ray, or MRI.

Second, this case demonstrates the profound impact that armed conflict can have on non-violent disease progression by disrupting local healthcare infrastructure. As highlighted by the Lancet in 2021, armed conflict disproportionately affects women and children, leading to increased morbidity and mortality not only from direct violence but also from malnutrition, physical injuries, infectious diseases, and chronic conditions [6]. For this patient, what should have been a straightforward diagnosis and referral for surgical treatment was delayed, leading to a significant deterioration in her condition. This delay risked malignant transformation and permanent damage to her dominant hand. Ensuring the continued operation of primary health centres in conflict zones is crucial for the timely diagnosis and referral of surgical conditions and to prevent such outcomes.

## Conclusion

We present the case of a large myxoma of the right hand in an internally displaced person from the DRC, a condition that was neglected due to the lack of adequate primary healthcare in a region affected by internal conflict. Despite a positive outcome with no malignant transformation or functional deficit, this case highlights the serious health consequences of disrupted healthcare infrastructure in conflict zones, where patients are often unable to access timely medical care.
